# Development of ReproKnow, a reproductive knowledge assessment for women with rheumatic diseases

**DOI:** 10.1186/s41927-019-0091-6

**Published:** 2019-10-21

**Authors:** Mehret Birru Talabi, Megan E. B. Clowse, Susan J. Blalock, Galen Switzer, Lan Yu, Alaina Chodoff, Sonya Borrero

**Affiliations:** 1 Division of Rheumatology and Clinical Immunology, Department of Medicine, University of Pittsburgh, 3500 Terrace Street, Pittsburgh, PA 15261 USA; 2 Duke University Medical Center, Durham, NC USA; 3 University of North Carolina Eshelman School of Pharmacy, Chapel Hill, NC USA; 4 Division of General Internal Medicine, Department of Medicine, University of Pittsburgh, Pittsburgh, PA USA; 5 Division of General Internal Medicine, University of Pittsburgh and UPMC, Pittsburgh, PA USA; 6 Veteran’s Affairs Pittsburgh Center for Health Equity Research and Promotion, Pittsburgh, PA USA

**Keywords:** Women’s health, Reproduction, Contraception, Autoimmune, Pregnancy

## Abstract

**Background:**

The objective of this study was to develop an assessment tool, ReproKnow, to evaluate the reproductive health knowledge of women with a wide range of rheumatic diseases.

**Methods:**

The 10-item multiple-choice questionnaire was developed with feedback from a panel of content experts and female patients with rheumatic diseases. Construct validity using known-groups analysis was evaluated through comparison of median total ReproKnow scores between rheumatology fellows and nurses. Female patients aged 18–50 years were recruited to take ReproKnow and demographic questionnaires in two outpatient clinics. Associations between patients’ mean total knowledge scores and demographic characteristics were assessed using independent-sample t-tests. Questions were also categorized by topical area, and the percentages were calculated.

**Results:**

The completion rate of questions in ReproKnow was 100% across all users. Median ReproKnow scores were significantly higher among rheumatology fellows than among nurses (*p* = 0.045). The 153 patients recruited to the study had at least one of 15 rheumatic diseases. Patients’ mean knowledge score was 5.05 (SD 2.24) out of a possible high score of 10. Patients who were younger, White, and more educated had significantly higher scores than did other patients (*p*’s < 0.05). Patients who bore children after their disease diagnosis had higher knowledge scores than did women whose children were born prior to their diagnosis; in contrast, women with histories of surgical sterilization or hysterectomy had lower knowledge scores than other women. Knowledge scores of women who used potentially fetotoxic medications did not vary from the remainder of the sample. Patients demonstrated gaps in knowledge about birth outcomes, contraceptive efficacy, and breastfeeding safety.

**Conclusions:**

Initial testing of ReproKnow suggests that it may be a promising tool to assess the reproductive health knowledge of women with diverse rheumatic diseases. Specific knowledge deficits elicited from ReproKnow may be important targets for future educational interventions.

## Background

Women with rheumatic diseases may face considerable reproductive health challenges during their childbearing years. Rheumatic diseases such as systemic lupus erythematosus (SLE), Sjogren’s syndrome, inflammatory arthritis, the inflammatory myopathies, and vasculitides have been associated with higher rates of pregnancy-associated mortality and morbidity compared to rates among healthy women [1–5]. Several common anti-rheumatic drugs have well-established teratogenic potential (e.g.*,* methotrexate, mycophenolate mofetil) [6, 7] and estrogen-containing contraceptive methods may increase the risk of thromboembolism among women with anti-phospholipid antibodies [8, 9].

It is important that women are empowered to make well-informed reproductive decisions that optimize their chances for favorable health outcomes. Such decisions may include planning their pregnancies for a time when their diseases are quiescent or adequately controlled on safe anti-rheumatic drugs, or by selecting non-hormonal birth control methods depending on their thrombotic risk— practices that have been shown to improve health outcomes, and that are supported by the American College of Rheumatology (ACR) and the European League Against Rheumatism [10, 11]. In contrast, women who lack knowledge about the relationships between their diseases and pregnancy outcomes may make less-informed health decisions that have deleterious clinical consequences. For example, a woman who erroneously believes that hormonal contraception is incompatible with her rheumatic disease may avoid using birth control, which could increase her risk for an unintended pregnancy that occurs while her disease is active or while using a fetotoxic anti-rheumatic drug.

Limited available research seems to suggest that some women with rheumatic diseases may lack adequate knowledge to make well-informed family planning decisions, which might eventually culminate in suboptimal reproductive health outcomes. Young women with rheumatoid arthritis (RA) in one qualitative study reported that they struggled to find reliable or relevant information about RA and pregnancy [12]. Young women with inflammatory arthritis in another study relied on unverified blogs, social media, and online forums to find relevant information about arthritis and pregnancy [13].

To develop tailored interventions that ameliorate important gaps in patients’ reproductive knowledge, those gaps in knowledge must first be identified. A number of self-administered questionnaires to assess patients’ knowledge have been developed across medical disciplines for conditions such as diabetes, inflammatory bowel disease (IBD), asthma, and osteoarthritis [14–17]. These questionnaires are generally paper-based, low-cost, and easy to administer in clinical or research settings. Self-administered questionnaires may minimize physician burden and increase efficiency by identifying patients’ specific knowledge gaps prior to the physician encounter; the physician may then educate patients on the knowledge gaps that are most pertinent to their medical conditions. Patients’ performance on self-administered assessments have been linked to health behaviors; for example, among young women who did not desire pregnancy, women with lower knowledge scores on a contraception self-administered questionnaire were found to have lower rates of contraception continuation at 6 months’ follow-up [18]. This suggests that such self-administered assessments have the potential to identify key gaps in patients’ knowledge that predict suboptimal health behaviors, and may be intervened upon to enhance their health outcomes.

Self-administered questionnaires may be particularly valuable for assessing reproductive health knowledge of women with rheumatic diseases in the clinical setting. Several survey-based studies suggest that women with rheumatic diseases infrequently receive reproductive health education or family planning counseling from their providers, even when their disease severity or use of teratogenic anti-rheumatic drugs increases their risk for pregnancy complications [19–22]. Providers who do attempt to clarify what patients know or do not know about their diseases and reproductive health may obtain a highly subjective assessment depending on the questions that they ask or do not ask, or their assumptions about the information that they believe is most relevant to the patient. An objective, standardized questionnaire that assesses multiple dimensions of women’s reproductive knowledge may increase the chances that a conversation occurs between providers and patients about key reproductive health issues that might be particularly relevant to the patient.

Few self-administered assessments exist for to assess reproductive health knowledge among women with rheumatic diseases. The Pregnancy in Rheumatoid Arthritis Questionnaire (PIRAQ) is a 17-item questionnaire used in research [23]. The Crohn’s and Colitis Pregnancy Knowledge Score (CCP-Know) is a 17-item questionnaire developed for clinical and research settings [24]. CCP-Know scores, in which higher scores indicated better knowledge, were found to be low in a sample of reproductive-age women with IBD, particularly among women who had not experienced a pregnancy after their disease diagnosis— and therefore might not be expected to have the experiential knowledge to answer the CCP-Know questions accurately.

The objectives of the current study were to: 1) develop a self-administered questionnaire (ReproKnow) to assess reproductive-age women’s knowledge of pregnancy-related issues in the rheumatic diseases; 2) evaluate the use of ReproKnow in a community-based cohort of women of reproductive age with rheumatic diseases, and describe patients’ reproductive knowledge.

## Methods

This study was approved by the University of Pittsburgh Institutional Review Board (PRO17080373).

### ReproKnow development

The content of ReproKnow reflected topics addressed in a patient educational pamphlet produced by the ACR about women’s reproductive health that is freely available on its website, and a review article about family planning for women with rheumatic diseases written by several of the current manuscript’s authors [25, 26]. Preliminary questions that addressed heritability of rheumatic diseases, birth outcomes, likelihood of fertility, contraception safety and efficacy, preconception care, pregnancy management, lactation/breastfeeding safety, and medication risk/safety, were developed by one of the principal investigators (M.B.T.). Content validation, as defined by Haynes et al., should include population and expert sampling for the initial generation of items and other elements of the scale [27]. To optimize the content validity of ReproKnow, a group of local and national rheumatologists, obstetrician-gynecologists, internists with formal women’s health sub-specialization, nurses, a pharmacist, and a survey methodologist, reviewed the questions. Based on their input, six questions were extracted, and the remaining questions were refined. Three female reproductive-age patients with SLE were recruited from an outpatient rheumatology clinic to participate in cognitive, “think-aloud” interviews while they used the preliminary tool. Their feedback about the clarity and content of the tool were used to make additional revisions to the questions and response options.

The current version of ReproKnow includes ten multiple-choice questions that assessed reproductive knowledge across a range of topical domains (Additional file 1). Each question has three to six answer choices, including a “Not Sure” option for all questions. Total, or overall, knowledge scores may range from 0 to 10, with 10 indicating a perfect score on the assessment. ReproKnow was scored at a third-grade reading level, according to the Flesch-Kincaid reading ease scale [28].

### Preliminary validation

Construct validity assesses the extent to which a scale reflects the abilities of different raters or users. We assessed one dimension of construct validity using a known-groups analysis to compare median total ReproKnow scores of rheumatology fellows to those of rheumatology nurses. We hypothesized that fellows would have higher scores as they have more formal medical training. Rheumatology fellows in their first or second years of training, and rheumatology nurses from two outpatient clinical practices, were invited to complete ReproKnow. Participation was voluntary and the tool was self-administered. Responses were returned anonymously; thus, individual subject characteristics were not collected.

Cronbach’s alpha was used to evaluate the internal consistency of ReproKnow; a coefficient of 0.7 or higher is generally considered to be acceptable for established scales, although coefficients of at least 0.6 may be considered for newly-created or preliminary scales [29–31]. We also completed a principal components analysis (PCA) for nominal level variables in order to assess the dimensionality of the scale, with a confirmatory factor analysis for dichotomous variables using the Hull method to determine the number of factors in the scale [32].

### Patient sample and data collection

We administered ReproKnow to female patients aged 18 to 50 years old who were established patients of either a community-based or an academic rheumatology practice affiliated with a large health care system in western Pennsylvania, U.S.A. Patients who had scheduled appointments during the study timeframe were pre-screened for eligibility based on age and gender. Eligible patients were approached by study coordinators immediately after their physician visits. Patients who agreed to participate and provided verbal consent were subsequently asked to complete a paper version of the ReproKnow questionnaire.

Women also completed a brief demographic survey, which included questions about age, race/ethnicity, education, disease diagnosis, and current use of biologic or non-biologic anti-rheumatic drugs. We were interested in assessing the reproductive histories of patients in the study to assess whether their knowledge scores might reflect prior experiences with childbearing. Therefore, we inquired about their numbers of biological children, the temporal associations between their childbearing and rheumatic diagnosis, whether their rheumatic diagnosis influenced their childbearing decisions, and if they had a history of surgical sterilization or hysterectomy.

Patients’ rheumatic diagnoses were elicited via free text responses, while the remainder of the demographic answer choices were presented in multiple-choice format. Patients were asked to select the anti-rheumatic drugs that they used from a list of commonly-used biologic and non-biologic disease-modifying anti-rheumatic drugs; we categorized medications as potentially teratogenic versus not teratogenic based on published international consensus guidelines [6, 7, 33].

### Statistical analyses

For construct validation testing, descriptive statistics were used to evaluate the knowledge scores within the rheumatology fellow and nurse groupings. The Kruskall-Wallis test for nonparametric data was used to compare scores between the fellows and nurses.

Among patients who completed ReproKnow, patients’ demographic answer choices were collapsed into two-category variables due to low cell counts for certain responses (i.e., age, race/ethnicity, educational attainment, type of sterilization procedure). Descriptive statistics were used to calculate patients’ total knowledge scores. Independent sample t-tests were used to evaluate relationships between patients’ demographic characteristics and their mean total knowledge scores. Patients’ correct responses to individual ReproKnow questions were also calculated. We also assessed the aggregate percentage of correct scores to the five medication risk questions among women who used potentially fetotoxic drugs versus other women in the sample.

Statistical analyses were performed using SPSS version 24, with two-tailed *p* < 0.05 signifying statistical significance. As one of our principal objectives was to generate hypotheses about the associations among knowledge, demographics, and reproductive profiles, we did not adjust for multiple comparisons.

## Results

### Subject characteristics

Construct validation testing was performed with rheumatology fellows and nurses; all six fellows (100%) and five of the six nurses (83.3%) who were invited to complete ReproKnow, chose to participate in the study.

To describe reproductive knowledge among patients, we also recruited and tested ReproKnow among 153 female patients. Patient subjects had a mean age of 38.3 years old (S.D. 8.2), most participants were white (77.0%), and half of the women had at least a college degree (50.0%). SLE (23.0%), Sjogren’s syndrome (15.0%), and RA (14.0%) were the most common diagnoses, but the sample also included women with undifferentiated connective tissue disease, psoriatic arthritis, spondyloarthritis, antiphospholipid antibody syndrome, inflammatory bowel disease, systemic sclerosis, inflammatory myositis, mixed connective tissue disease, ANCA vasculitis, Takayasu arteritis, Bechet’s, and polyarteritis nodosa. Some women did not report their rheumatic diagnosis (*n* = 12) or only reported a non-rheumatic disease diagnosis (*n* = 22; e.g., fibromyalgia, unspecified muscle or joint pain, or positive blood test, such as an anti-nuclear antibody). We conducted a sensitivity analysis to evaluate if results differed when we excluded these 34 women from the sample. The results did not change, and as some of the women may have had a rheumatic disease that they chose not to report, or a disease that had not yet been formally diagnosed, we elected to retain all participants in the final sample.

We also assessed the reproductive histories of women in our sample. Three patients were pregnant at the time that they completed ReproKnow. A majority of women had at least one child (64.1%), and most women experienced all of their pregnancies prior to diagnosis of their rheumatic diseases (75.8%). Approximately 27% of women reported that their disease had affected their decision to have any or additional children.

Over one-quarter (25.5%) of women reported current use of at least one anti-rheumatic drug with fetotoxic potential, which in this sample, included methotrexate, leflunomide, and mycophenolate mofetil [6].

### Construct validity

The completion percentage for all of the questions in ReproKnow was 100% for rheumatology fellows and nurses. The median total knowledge score for fellows was 8.5 (mean 8.33, S.D. 1.21, range 7–10), and was 7.0 for nurses (mean 6.8, S.D. 0.45, range 6–7). As scores were not normally distributed, nonparametric testing was used to compare median scores between these groups; fellows’ scores were significantly higher than were nurses’ scores (*p* = 0.045).

### Internal consistency

The Cronbach’s alpha statistic for ReproKnow was based on patient responses, and was estimated at 0.62, which demonstrates moderate internal consistency. To assess if the moderate internal consistency was secondary to multidimensionality in the scale, we subsequently conducted a principal components analysis (PCA) for nominal level variables. The PCA identified four items on two dimensions that had no clinically meaningful relationships. We next removed these four items from the analysis, and subsequently removed all possible combinations of the items from the analysis. This sub-analysis did not change the alpha level. Our factor analysis results were similar to the PCA analysis in that a one-factor solution was also recommended using Hull criteria, but that factor explained a minority of the overall variance (37.6%) and several items had either moderately low (<.40, item 4; <.45 items 7 and 9), or very low (< .20, item 3; <.25, item 5) loadings.

### Patients’ total reproductive knowledge scores

**Fig. 1:**
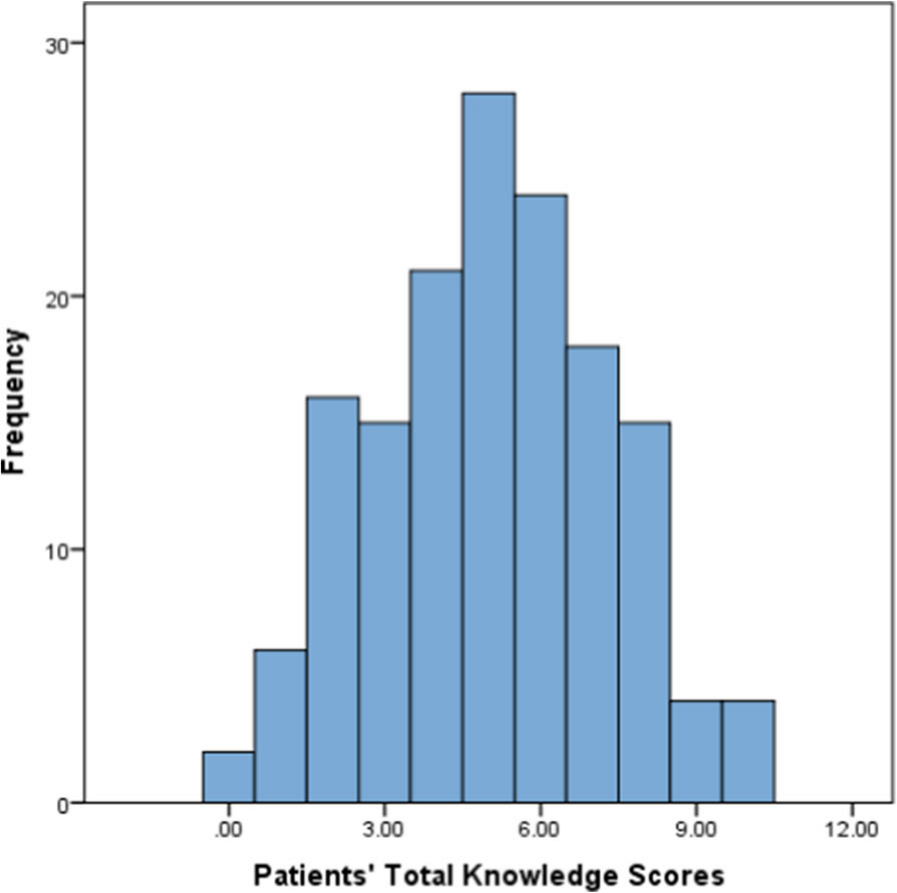
Distribution of Patients’ Total Knowledge Scores. Legend: *N* = 153; Mean score: 5.05; Standard deviation: 2.244

The completion percentage for all of the questions in ReproKnow was 100% for patients. Patients’ total reproductive knowledge scores ranged from 0 to 10, with a median score of 5.0 and mean score of 5.05 (SD 2.24). Patient scores were normally distributed (Fig. 1).

Table 1 presents the associations between patients’ characteristics and mean knowledge scores. Younger women had significantly higher total knowledge scores than did older women, Whites had higher scores than non-Whites, and highly-educated women had higher scores than did women with less education (*p*’s < 0.05). Total knowledge scores did not differ between women who used potentially teratogenic medications and those who used safer medications.

**Table 1:** Patient Demographics and Characteristics and ReproKnow Total Scores

Characteristics	N (%)	Total Score Means (S.D.)	Mean Score Difference	*P* value
Age (years)				
**18–34**	46 (30.1)	5.63 (2.22)	0.83	0.037
35–50	106 (69.3)	4.80 (2.23)		
Race				
**White**	118 (77.1)	5.45 (2.06)	1.8	0.002
Non-White	34 (22.2)	3.67 (2.37)		
Education				
**College/Graduate School**	76 (49.7)	5.61 (5.62)	1.13	0.002
< High School/Some College	76 (49.7)	4.48 (2.21)		
Current Use of Potentially Teratogenic Medication				
**Yes**	39 (25.5)	5.10 (2.37)	0.068	0.42
No	114 (74.5)	5.03 (2.21)		
Pregnancy After Diagnosis				
**Yes**	23 (24.2)	6.00 (2.28)	1.16	0.015
No	72 (75.8)	4.83 (2.16)		
Disease affected decision to have more children				
**Yes**	42 (28.6)	5.19 (2.32)	0.11	0.19
No	105 (71.4)	5.08 (2.07)		
Prior Sterilization or Hysterectomy Procedure				
**Yes**	40 (26.1)	4.25 (1.97)	- 1.09	0.008
No	112 (73.2)	5.34 (2.28)		

Mean score difference was calculated by subtracting score mean of bolded group from the score mean of un-bolded group

Among parous women in the sample, those patients who were either currently pregnant or who had a child born after their rheumatic diagnosis (*n* = 23 for combined group) had higher knowledge scores than did women whose children were born prior to their disease diagnosis (*n* = 72) (*p* = 0.015). Women who had prior sterilization or hysterectomy procedures had lower total knowledge scores than women who did not have these procedures (*p* = 0.008).

### Assessment of patients’ reproductive knowledge by topic

Patients’ knowledge scores categorized by topical domains are presented in Table 2. Most patients correctly answered questions about heritability (75.8%) and pregnancy management (63.8%), whereas fewer patients correctly answered questions about birth outcomes (30.8%) and breastfeeding safety (28.8%). Approximately 50% of respondents correctly answered questions about fertility, contraception, preconception planning, and medication risk.

**Table 2:** Percentage of Patients’ Correct Answers By Question and By Concept Area (Mean Percentage (%)

	% Correct
Heritability	
1. Moms with autoimmune diseases pass their diseases on to their children (A^a^: Sometimes)	75.8
Birth Outcomes	
1. If I have an autoimmune disease, my baby’s chances of being born with a birth defect are (A: Low)	32.7
2. If I am pregnant and have a disease flare, my baby may be: (A: Born too early)	28.8
Fertility	
1. Most women with autoimmune diseases can get pregnant as easily as other women (A: Yes)	51.6
Contraception	
1. Can most women with autoimmune diseases use birth control safely? (A: Yes)	64.7
2. Which type of birth control is the best at preventing pregnancy? (A: Intrauterine device (IUD))	41.2
Preconception Planning	
1. When is the best time for a woman with an autoimmune disease to get pregnant? (A: After her disease is controlled on safe meds for a few months)	54.2
Pregnancy Management	
1. If I find out that I’m pregnant, what should I do next? (A: Continue my meds until I talk with my doctor)	75.2
2. If I am pregnant and have a flare of my disease (A: I may need to use meds to protect me and my baby)	52.3
Breastfeeding	
1. Moms with autoimmune diseases who are on safe meds (A: Usually can breastfeed safely, Make breastmilk that is nutritious as other women’s)	28.8
Medication Risk	
1. Can most women with autoimmune diseases use birth control safely? (A: Yes)	64.7
2. When is the best time for a woman with an autoimmune disease to get pregnant? (A: After her disease is controlled on safe meds for a few months)	54.2
3. If I find out that I’m pregnant, what should I do next?(A: Continue my meds until I talk with my doctor)	75.2
4. If I am pregnant and have a flare of my disease (A: I may need to use meds to protect me and my baby)	52.3
5. Moms with autoimmune diseases who are on safe meds (A: Usually can breastfeed safely, Make breastmilk that is nutritious as other women’s)	28.8

Multiple topical domains may be covered by a single question, thus questions may appear more than once in table

^a^A = Correct Answer

Among women who used potentially fetotoxic anti-rheumatic drugs, 54.4% of questions about medication risk were answered correctly; 55.2% of these same questions were answered correctly by women who did not use fetotoxic drugs.

## Discussion

The ReproKnow tool was designed to evaluate what women with a broad range of rheumatic diseases know about disease-related reproductive health topics. In our cohort of female, reproductive-age patients, ReproKnow revealed key knowledge gaps related to birth outcomes, safety of lactation, likelihood of fertility, efficacy of contraceptive methods, and medication safety.

The content of ReproKnow was intended to be relevant for reproductive-age women with any rheumatic disease that is treated by a rheumatologist. Several reproductive knowledge assessments exist for specific immune-mediated diseases (e.g., Pregnancy in Rheumatoid Arthritis Questionnaire (PIRAQ) [23], and Crohn’s and Colitis Pregnancy Knowledge Score (CCP-Know) [24]. However, such tools are not available for the majority of rheumatic diseases, including diseases with high pregnancy-associated mortality and morbidity, such as systemic lupus erythematosus. This underscores the potential utility of a general tool that assesses knowledge about the shared reproductive risks across rheumatic diseases. ReproKnow is also brief, readable, inexpensive, and easy to administer, and may be adaptable for a wide range of research or clinical purposes. The 100% completion rate among all users underscores its feasibility, particularly in clinical settings.

Our study provides evidence that ReproKnow is a promising tool for assessing reproductive knowledge. First, knowledge scores reflected the level of formal rheumatology training and education of users, including fellows, nurses, and patients, which suggests that it might have acceptable construct validity. Secondly, ReproKnow appeared to reflect patients’ knowledge based on their reproductive experiences. Women who had children after their disease diagnosis had higher knowledge scores than did other women; these women likely had some disease-related health counseling during their pregnancies, which may have translated into greater reproductive knowledge. Similarly, lower knowledge scores attained by women who had hysterectomies or sterilization procedures may be reflective of less reproductive health counseling given to women who do not have reproductive potential.

ReproKnow’s relatively low internal consistency might be considered a potential weakness. While Cronbach’s alpha is ideal for scales that have multiple response options (e.g., Likert), coefficients may be artificially low for scales with fewer responses [29]. Our interpretation of our findings from PCA and factor analysis were that a single factor solution did not sufficiently explain the variance in the model, and a multiple-factor solution lacked clinical or conceptual meaning. It is possible that future research with ReproKnow involving larger samples of women will reveal a meaningful latent structure. However, the internal consistency, PCA, and factor analysis results may also reflect that ReproKnow is meant to test a broad range of topics across reproductive health, including pregnancy, pregnancy prevention, lactation, and heritability.

Certain patient characteristics were associated with better total knowledge scores, including younger age, White race, and higher educational attainment. However, total knowledge scores or scores on the medication risk questions did not differ between users or non-users of potentially fetotoxic medications. Gaps in reproductive health knowledge among women who use fetotoxic medications may have particularly deleterious effects, especially among women who conceive while using these drugs.

Our analysis also assessed women’s knowledge about specific reproductive health domains. Most women overestimated their offspring’s risk for congenital anomalies and underestimated the safety of breastfeeding. This finding has been previously reported in studies of the general population, in which many women overestimate the absolute risk of congenital fetal anomalies, and women who use medications for any indication are less likely to breastfeed due to concerns about safety [34, 35]. However, among women with rheumatic diseases, the risk of congenital anomalies does not differ significantly from the general population, including among children who have been exposed to pregnancy-compatible anti-rheumatic drugs before or during pregnancy [36]. Breastfeeding also appears to be safe for women who use lactation-compatible medications [33]. Our results suggest that some patients may benefit from counseling about risk of congenital anomalies and breastfeeding safety, to help them to make informed decisions about childbearing and breastfeeding.

Approximately half of patients incorrectly answered questions about fertility, the efficacy and safety of contraceptive methods, and preconception planning; these knowledge gaps may affect reproductive decision-making and behaviors, and translate into suboptimal reproductive health outcomes. Work by Mosher et al. suggests that women who underestimate their childbearing potential may be more likely to engage in unprotected sex, thus increasing their risk of unintended pregnancy [37]. In our cohort, only 41.2% of women were able to correctly identify the most effective contraceptive method of the choices provided. Women with low contraception knowledge may also overestimate the efficacy of methods such as condoms as compared to more efficacious methods (e.g. intrauterine devices), which might further increase their risk of unintended pregnancy even if they do use contraception [38]. More work is needed to assess whether patients with better reproductive knowledge more accurately ascertain reproductive risks associated with their diseases and medications, and make more informed family planning decisions. Several consensus guidelines and reviews are available to help providers educate patients with rheumatic diseases about reproductive health and family planning [11, 33, 39–41].

Our study and analytic design had certain limitations. First, while educational attainment and proportion of white participants in our sample were similar to the demographics of the general western Pennsylvania population, the generalizability of our findings to women from other racial/ethnic or socioeconomic backgrounds may be limited [42]. Our findings may also overestimate the reproductive knowledge of women with rheumatic diseases: women with low functional literacy may have declined participation, and white women, whose knowledge scores were generally higher than other women, were overly represented in our cohort. Therefore, knowledge scores in this cohort may be higher than scores in a more diverse group of women with rheumatic diseases. In addition, while ReproKnow asked women to answer questions based on “most women’s” experiences, some women may have answered questions based on their own experiences; for example, women who personally experienced contraceptive failure or infertility, might have answered those questions incorrectly based on their own experiences rather than an understanding of population risk. Our perspective is that a “wrong answer” might actually provide an opportunity for a provider to clarify patients’ myths or misconceptions.

In addition, more research is needed to further develop the psychometric properties of ReproKnow. Additional testing of the tool in a variety of clinical (e.g., community-based, academic, or hospital settings, and different geographic locations) or research settings will help to further support the validity and reliability of ReproKnow. Our sample was not racially diverse, and the tool should be explored in more diverse populations of women with rheumatic diseases, perhaps with a wider range of rheumatic diseases. Criterion validity could be explored by assessing whether high scores on ReproKnow translate to better reproductive outcomes over time, perhaps in a longitudinal cohort of women with rheumatic diseases. High scores on a self-administered contraception knowledge assessment in one study predicted more consistent contraception use over time among young women who did not desire pregnancy [18]; thus, it is conceivable that patients’ reproductive knowledge could be linked to behaviors that optimize patient’s reproductive outcomes. This should be explored in future testing of ReproKnow.

In conclusion, ReproKnow is a tool that may help to evaluate the reproductive knowledge of women with a range of rheumatic diseases across a variety of topical domains. Women who use potentially fetotoxic medications appear to be a particularly important target for educational interventions. Given the particularly low scores in contraception, breastfeeding, and birth outcomes, women also may benefit from enhanced knowledge about these topics. Providers should consider identifying addressing specific knowledge gaps in order to provide women with rheumatic diseases with patient-centered, comprehensive care. More research is needed to determine what types of educational interventions may help to close the knowledge gaps in this high-risk population of women.

## Conclusions

The purpose of this study was to develop and test a reproductive knowledge assessment for women with rheumatic diseases, a clinically vulnerable group. Our preliminary validation suggests that ReproKnow has the potential to expose important reproductive health knowledge gaps among these patients. The extent to which these gaps in knowledge predispose to adverse reproductive outcomes should be studied in the future. Additional validation testing should also be conducted in different populations of women with rheumatic diseases.

## Supplementary information

**Additional file 1.** ReproKnow instrument.

## Data Availability

Most data and materials are included in this published article and its supplementary files. All other data and materials for this study may be obtained by contacting the corresponding author (birrums@upmc.edu).

## Abbreviations

ACR: American College of Rheumatology
APS: Antiphospholipid antibody syndrome
CCP-Know: Crohn’s and Colitis Pregnancy Knowledge Score
IBD: Inflammatory bowel disease
PCA: Principal components analysis
PIRAQ: Pregnancy in Rheumatoid Arthritis Questionnaire
RA: Rheumatoid arthritis
SLE: Systemic lupus erythematosus
