# Supplementary material for: Development of ReproKnow, a reproductive knowledge assessment for women with rheumatic diseases
Source: BMC Rheumatol. 2019 Oct 21;3:40. doi: 10.1186/s41927-019-0091-6 (PMC6805554; doi:10.1186/s41927-019-0091-6)
Supplement: Supplementary file 1 — Additional file 1. ReproKnow instrument. [file 41927_2019_91_MOESM1_ESM.docx]

There are 10 questions. Please circle your answer.

All questions have 1 best answer except #10, which may have more than 1 best answer.

Autoimmune diseases include most of the diseases that are treated in this clinic.

Questions:

1. Moms with autoimmune diseases **pass their diseases on to their children**
   1. Always
   2. Sometimes
   3. Never
   4. Not sure

2. If I have an autoimmune disease, my **baby’s chances of being born with a birth defect** are

1. High
2. Low
3. Not sure

3. Most women with autoimmune diseases **can get pregnant** as easily as other women

a. Yes

b. No

c. Not Sure

4. Can most women with autoimmune diseases **use birth control safely**?

a. Yes

b. No

c. Not sure

5. Which type of birth control is the **best at preventing pregnancy**?

a. IUD (intrauterine device)

b. Depo-Provera (“the shot”)

c. Condoms

d. Withdrawal (“pull-out”)

e. They are all equally good at preventing pregnancy

f. Not sure

6. When is the **best time** for a woman with an autoimmune disease **to get pregnant**?

1. As soon as she can
2. After she has stopped all of her meds for a few months
3. After her disease is controlled on safe meds for a few months
4. Never
5. Not sure

7. **If I find out that I’m pregnant**, what should I do next?

a. Stop all my meds

b. Continue my meds until I talk with my doctor

c. Use the Internet to figure out what meds are safe for me to use

d. Call my doctor to schedule my first ultrasound

e. Not sure

8. If I am **pregnant and have a flare** of my disease

a. My baby will be fine, but I can get sick

b. I may need to use meds to protect me and my baby

c. I should not be treated with meds because it can hurt the baby

d. Not sure

9. If I am pregnant and have a disease flare, **my baby may be:**

a. Born with birth defects

b. Born too late

c. Born too early

d. Not sure

10. Moms with autoimmune diseases who are on **safe meds (Pick all that apply, may be more than 1 answer)**

1. Never should breastfeed
2. Can pass their disease to their babies through breast milk
3. Usually can breastfeed safely
4. Do not have breastmilk that’s as healthy as other women’s
5. Make breastmilk that is as nutritious as other women’s
6. Not sure
